# SARS-CoV-2 infection induces activation of ferroptosis in human placenta

**DOI:** 10.3389/fcell.2022.1022747

**Published:** 2022-11-08

**Authors:** Bingbing Wang, Wei-Bin Shen, Peixin Yang, Sifa Turan

**Affiliations:** Department of Obstetrics, Gynecology and Reproductive Sciences, University of Maryland School of Medicine, Baltimore, MD, United States

**Keywords:** SARS-CoV-2, positive-sense strand, ferroptosis, ACSL4, placenta, adverse pregnancy outcomes

## Abstract

Ferroptosis, a regulated non-apoptotic form of cell death, has been implicated in the response to varied types of infectious agents including virus. In this study, we sought to determine whether SARS-CoV-2 infection can induce activation of ferroptosis in the human placenta. We collected placentas from 23 pregnant females with laboratory-confirmed SARS-CoV-2 following delivery and then used RNA *in situ* hybridization assay for detection of viral positive-sense strand (PSS) to confirm that these placentas have been infected. We also used immunohistochemistry assay to assess expression levels of acyl-CoA synthetase long-chain family member 4 (ACSL4), an essential executioner of ferroptosis in the same specimens. Our results showed that ACSL4 expression was significantly increased in the group with positive positive-sense strand staining compared to their negative counterparts (*p* = 0.00022). Furthermore, we found that there was a positive trend for increased PSS staining along with increased ACSL4 expression. Our study supports that ferroptosis is activated in the response to SARS-CoV-2 infection in the human placenta, highlighting a molecular mechanism potentially linking this coronavirus infection and pathogenesis of adverse pregnancy outcomes.

## Introduction

Pregnancy represents a distinctive immunological condition and the remarkable changes in levels of both hormones and immune cell-released cytokines may enhance pregnant females’ vulnerability to viral infection including severe acute respiratory syndrome coronavirus 2 (SARS-CoV-2) (Alberca et al., 2020). Indeed, pregnant females are at increased risk for such coronavirus infection, and in turn, worse outcomes of coronavirus disease 2019 (COVID-19) (Narang et al., 2020). Multiple studies have reported higher rates of pregnancy-related complications, including preterm birth, preeclampsia, and stillbirth with SARS-CoV-2 infection during pregnancy (Garovic et al., 2020; Ganor Paz et al., 2021; Giesbers et al., 2021; Papageorghiou et al., 2021; Dubucs et al., 2022), warranting urgency to a pursuit of research into underlying molecular pathways leading to the pathogenesis of these adverse pregnancy outcomes and development of optimal COVID-19 treatment and preventive approaches during pregnancy.

The majority of adverse obstetrical consequences is embedded with a placental origin, which is associated with placental inflammation because of infectious or non-infectious causes (Ilekis et al., 2016). The human placenta synthesizes a large and diverse number of hormones and cytokines that exert major influences on ovarian, uterine, mammary, and fetal physiology, and there is good evidence supporting that placental dysfunction plays a key role in contributing to these common interrelated disorders, which occur in approximately 10%–20% of pregnancies (Lane-Cordova et al., 2019). The spectrum of placental pathological findings in pregnant females with SARS-CoV-2 infection showed substantial variability, but typically including chronic histiocytic intervillositis and syncytiotrophoblast necrosis, a form of cell injury-caused premature cell death in living tissues due to autolysis (Schwartz and Morotti, 2020).

Recent advances in understanding cell death under both physiological and non-physiological conditions have witnessed a significant shift with recognition of the role of ferroptosis in the regulation of cell fate in response to oxidative stress (Stockwell, 2022). Ferroptosis, a form of nonapoptotic programmed cell death, is characterized by redox-active iron-dependent hydroxy-peroxidation of polyunsaturated fatty acid (PUFA)-containing phospholipids and loss of lipid peroxidation repair capacity (Dixon et al., 2012). This unique form of lipotoxic cell death has been implicated in various human diseases, including heart and brain injury, cancer, asthma, and placental physiology (Stockwell, 2022). Furthermore, a number of studies have indicated lipotoxicity at a remarkably elevated level during the pathophysiological course of major pregnancy-associated disorders such as preeclampsia, fetal growth restriction, and prematurity (Beharier et al., 2021).

There is an increasing body of evidence supporting that placental SARS-CoV-2 infection is tightly associated with adverse pregnancy outcomes. While there are varied types of qualitative and quantitative techniques including RT-PCR, immunological assay, and viral genome sequencing used to confirm its presence in the human tissues (Rotondo et al., 2022), NICHD and Human Development SARS-CoV-2 Placental Infection Workshop have recently set up the criteria for determining placental SARS-CoV-2 infection with use of RNA *in situ* hybridization (RNA-ISH) to detect viral positive-sense strand (PSS) as the top approach defining the placental presence by SARS-CoV-2 virions (Roberts et al., 2021).

Iron overload in cells and tissues coupled with hyperferritinemia has been well documented in patients with severe SARS-CoV-2 infection (Cavezzi et al., 2020; Edeas et al., 2020; Fratta Pasini et al., 2021; Cavezzi et al., 2022), suggesting that the dysregulation of iron metabolism may play an important role in contributing to the pathogenesis of COVID-19. Indeed, while iron constitutes an essential element for all living cells, extra iron can cause overproduction of reactive oxygen species and damage of cellular macromolecules including lipids, nucleic acids, and proteins, thus leading to ferroptosis and multiorgan failure (Habib et al., 2021). In this study, we sought to investigate whether SARS-CoV-2 virus can infect human placenta from pregnant females tested positive for SARS-CoV-2 with use of RNA-ISH assay. Meanwhile, we employed immunohistochemical (IHC) staining assay to determine the placental abundance of acyl-CoA synthetase long-chain family member 4 (ACSL4), the key executioner of ferroptosis (Doll et al., 2017; Jiang et al., 2021; Stockwell, 2022). Finally, we analyzed correlation between PSS staining intensity and ACSL4 expression levels within the same specimens.

## Materials and methods

### Study patients

Patients with 18 years and older with laboratory-confirmed SARS-CoV-2 infection that delivered at University of Maryland Medical Center whose respective placental specimens have been sent for histological examination were included in the study. Patients with a history of adverse pregnancy outcomes, hypertension, diabetes, autoimmune diseases, and infectious diseases including human immunodeficiency virus and tuberculosis were excluded. Upon delivery, each placenta sample was coded with a 5-digital number. We also collected placentas from six normal patients tested negative for SARS-CoV-2 infection as the control. This study was approved by the Institutional Review Board of the University of Maryland, Baltimore, under the protocols HP-00091334 and HP-00100457.

### RNA-ISH

We conducted RNA-ISH in formalin-fixed paraffin-embedded (FFPE) slides with the use of the RNAScope 2.5 HD Detection (RED) Kit (ACD, CA) according to the protocol provided by the manufacturer as well as described in our recent publication (Liu et al., 2020; Shen et al., 2022b). Briefly, after deparaffinization and antigen retrieval, the slides were hybridized with the 40-ZZ probe targeting positive-sense strand (PSS), V-SARS-CoV-2-S (Cat#: 854841, ACD) in the oven at 40°C for 2 h. We then washed the slides with one X Wash Buffer for 2 min twice. The remaining hybridization procedure at 40°C included: 1) Amp 1, 30 min; 2) Amp 2, 15 min; 3) Amp, 30 min; 4) Amp 4, 15 min; and 5) Amp 5, 30 min and 6) Amp 6, 15 min under room temperature. Subsequently, the signals were detected by incubating with a mixture of RED-B and RED-A at a ratio of 1:60 for 10 min at room temperature, followed by counterstaining in 50% hematoxylin for 2 min.

The RNA-ISH was quantified with the use of QuPath, open-source software for digital pathology image analysis (Bankhead et al., 2017). The percentage of staining area by viral PSS RNA in the chorionic villi tissues, including syncytiotrophoblast, cytotrophoblast, and fetal blood vessels was normalized to nuclear hematoxylin staining intensity, and the mean was obtained from three random fields in the same cross-section. We set the overall scores ranging from 0 to four based on the percentage staining area. If the staining area is <0.1% the score was 0. Scores 1, 2, 3, and 4 was defined if the staining area percentage were ≥0.1 and <1%, ≥1 and <2%, ≥2 and <3%, ≥3%, respectively.

### IHC staining

IHC staining was performed as described in our recent studies (Shen et al., 2022a; Yao et al., 2022). Briefly, following deparaffinization and antigen retrieval, FFPE slides were blocked in phosphate-buffered saline (PBS) containing 4% donkey serum and 0.2% Triton X-100 for 1 h at room temperature. Then FFPE slides were incubated with primary antibody to ACSL4 at a dilution of 1:250 (Abcam, MA) overnight at 4°C; the sections were washed with PBS three times and incubated with biotinylated secondary donkey anti-rabbit antibody (Vector Laboratories, CA) in PBS with 0.2% Triton X-100 at a dilution of 1:500 for 1 h. Sections were washed again, and the secondary antibodies were detected by use of a DAB detection kit (Vector Laboratories), followed by counterstaining in 50% hematoxylin for 2 min.

ACSL4 staining in IHC was quantified with use of ImageJ, an image processing program developed at National Institutes of Health (Schneider et al., 2012). The mean of percentage of staining area by ACSL4 protein in the chorionic villi tissues was acquired from three random fields. We set the overall scores from 0 to nine based on the percentage staining area of <0.1%, ≥0.1 and <1%, ≥1 and <2%, ≥2 and <3%, ≥3% and <4%, ≥4 and <5%, ≥5 and <6%, ≥6 and <7%, ≥7 and <8%, and ≥9%, respectively.

### Statistical analysis

Quantitative comparisons of ACSL4 abundance between two groups were examined with the Mann-Whitney *U* test. We used the linear regression to analyze correlation between staining degree of PSS and ACSL4.

## Results

### ACSL4 expression increased in SARS-CoV-2-infected placenta compared to negative control

ACSL4 plays a critical role in induction of ferroptotic cell death in a variety of cell types including neurons, fibroblast, and vascular and immune cells (Yuan et al., 2016; Doll et al., 2017; Stockwell et al., 2017). By shaping the cellular lipidome, ACSL4 dictates cells’ sensitivity or resistance in response to ferroptosis. Enzymatically, ACSL4 catalyzes esterification of CoA to free fatty acids; subsequently, the formation of acyl-CoA induces oxidation and lipid biosynthesis of corresponding fatty acids.

**FIGURE 1:**
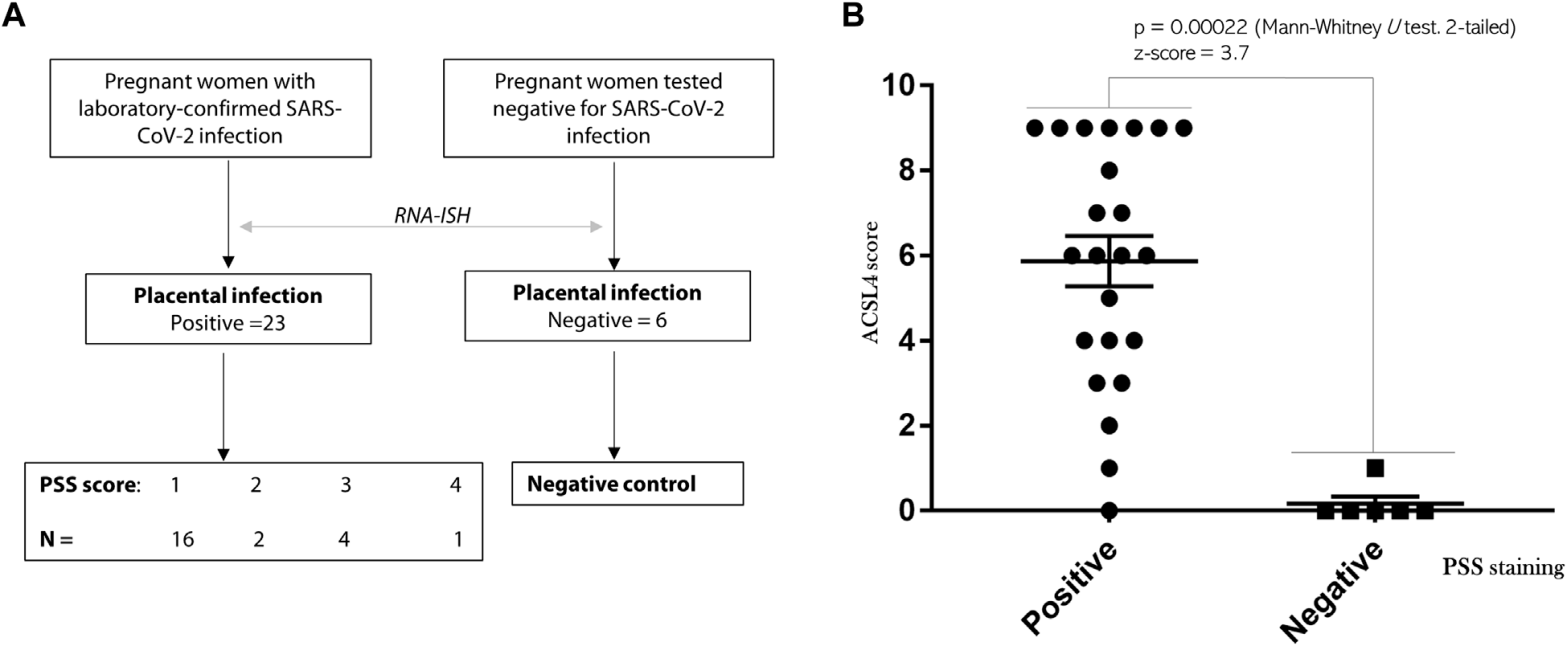
SARS-CoV-2 infection activates ACSL4 expression of placental origin. **(A)** The overview and PSS staining results of this study. A total of 23 patients with laboratory-confirmed SARS-CoV-2 infection were enrolled, which was followed by RNAScope-based RNA-ISH analysis to confirm viral presence in the placenta. Six placentas collected from patients test negative infection were used as the control. **(B)** Mann-Whitney *U* test was used to compare expression levels of ACSL4 between the group of positive (N = 23) and negative (N = 6) PSS staining. *p* = 0.00022. Z-score = 3.7.

The overview of this study design and results of placental SARS-CoV-2 infection are depicted in Figure 1A. We used RNAScope-based RNA-ISH assay for detection of PSS of this coronavirus, which provides the most rigorous evidence to define whether the placenta has been infected (Roberts et al., 2021). Among the 23 patients determined with positive staining of PSS in the placenta, there were a total of 16, 2, 4, and 1 cases that were scored at 1, 2, 3, and 4, respectively. We also used IHC to assess the expression levels of ACSL4 in the same specimens. The scores for ACSL4 expression in the positive PSS staining group ranged from 0 to 9, in contrast to only one weakly positive staining of ACSL4 detected in the negative control group. The nonparametric Mann-Whitney *U* test determines that there was a significant difference in ACSL4 expression existing between these two groups with a *p*-value of 0.00022 and z-score of 3.7 (Figure 1B).

Taken together, these results support that SARS-CoV-2 infection leads to activation of ferroptosis in the human placenta.

### Staining profile of SARS-CoV-2 and ACSL4 in the human placenta

**FIGURE 2:**
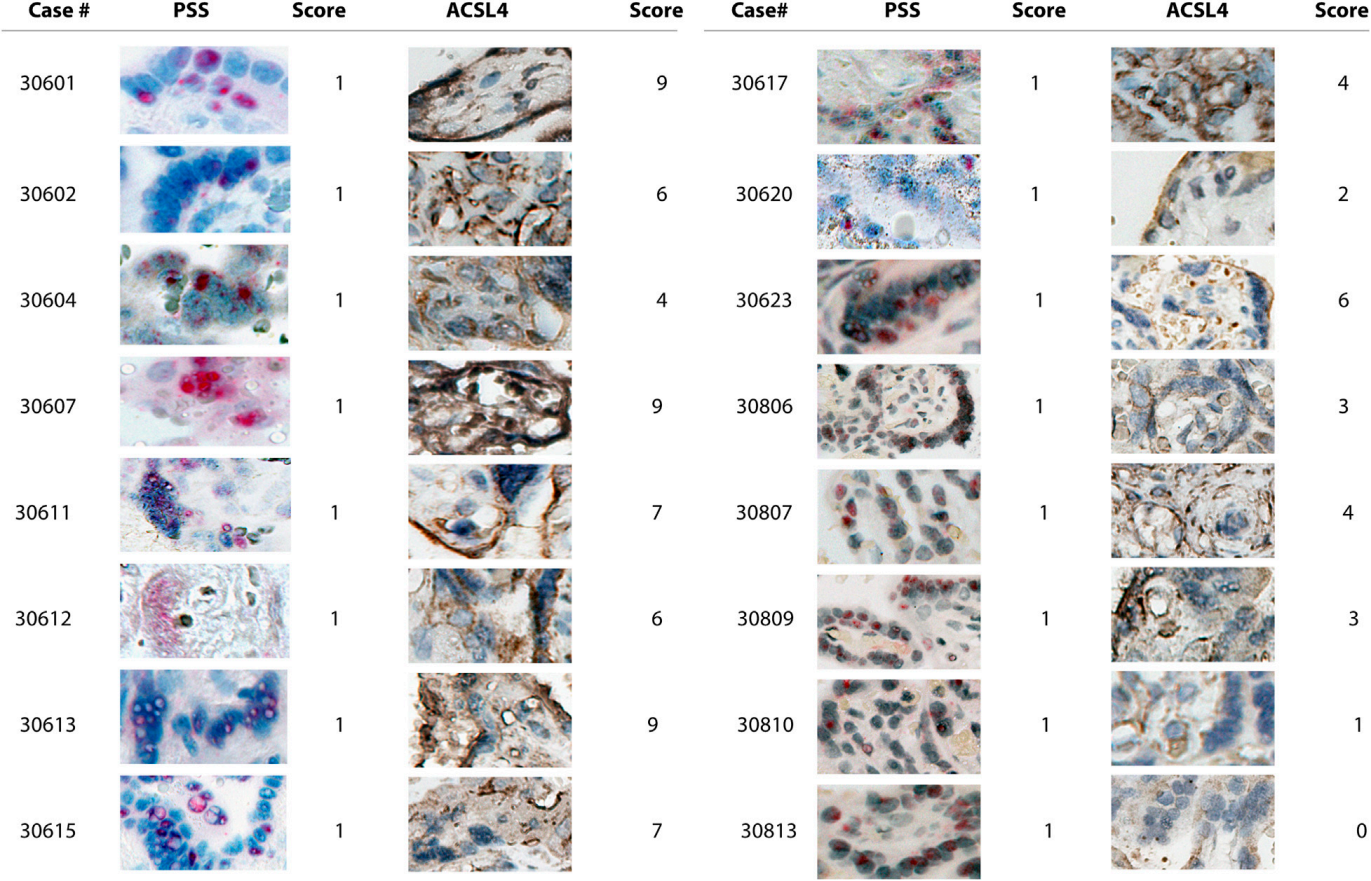
Microscope images (20X) of viral RNA and ACSL4 protein staining in placental villous tissues among specimens with PSS staining scored 1. RNA-ISH with use of RNAScope HD-RED kit and immunohistochemical staining (IHC) were performed to determine the abundance of the viral RNA genome and ACSL4 protein, respectively. The quantification and scoring criteria are detailed in Materials and Methods.

**FIGURE 3:**
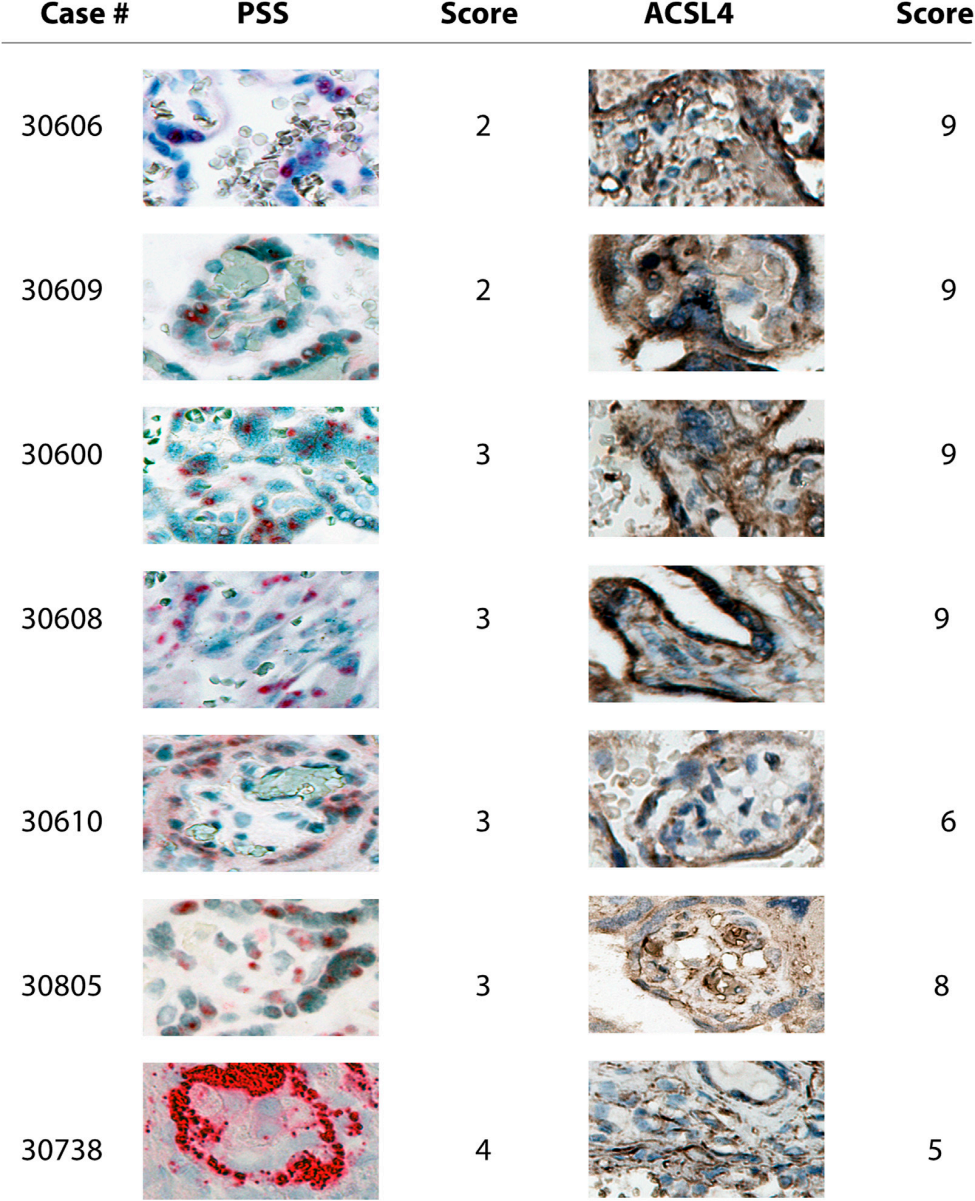
Microscope images (20X) of viral RNA and ACSL4 protein staining in placental villous tissues among specimens with PSS staining scoring ranged from two to 4. RNA-ISH with use of RNAScope HD-RED kit and immunohistochemical staining (IHC) were performed to determine the abundance of the viral RNA genome and ACSL4 protein, respectively.

We found that PSS staining was predominantly localized within the outermost syncytial knots/syncytiotrophoblast layer within the chorionic villi tissues in all the positive cases, independent of their respective scoring, consistent with that the placental barrier constitutes the first line of defense to protect the fetus from maternal viral infection (Figures 2, 3). Furthermore, PSS staining was also detected in the discontinuous monolayer of cytotrophoblast in some cases, *e.g*. #30600 and #30615 (PSS score = 1), #30609 (PSS score = 2), and #30600 (PSS score = 3), suggesting that this viral infection has crossed the placental barrier. Noticeably, in #30738 (PSS score = 4), fetal blood vessels demonstrated an exceedingly strong PSS staining, and our recent study has confirmed that this is the case of transplacental vertical transmission (Shen et al., 2022b).

Consistent with the results of PSS staining as described above, ACSL4 expression was primarily observed in the syncytiotrophoblast with some staining detected in cytotrophoblast at varying extents (Figures 2, 3).

**FIGURE 4:**
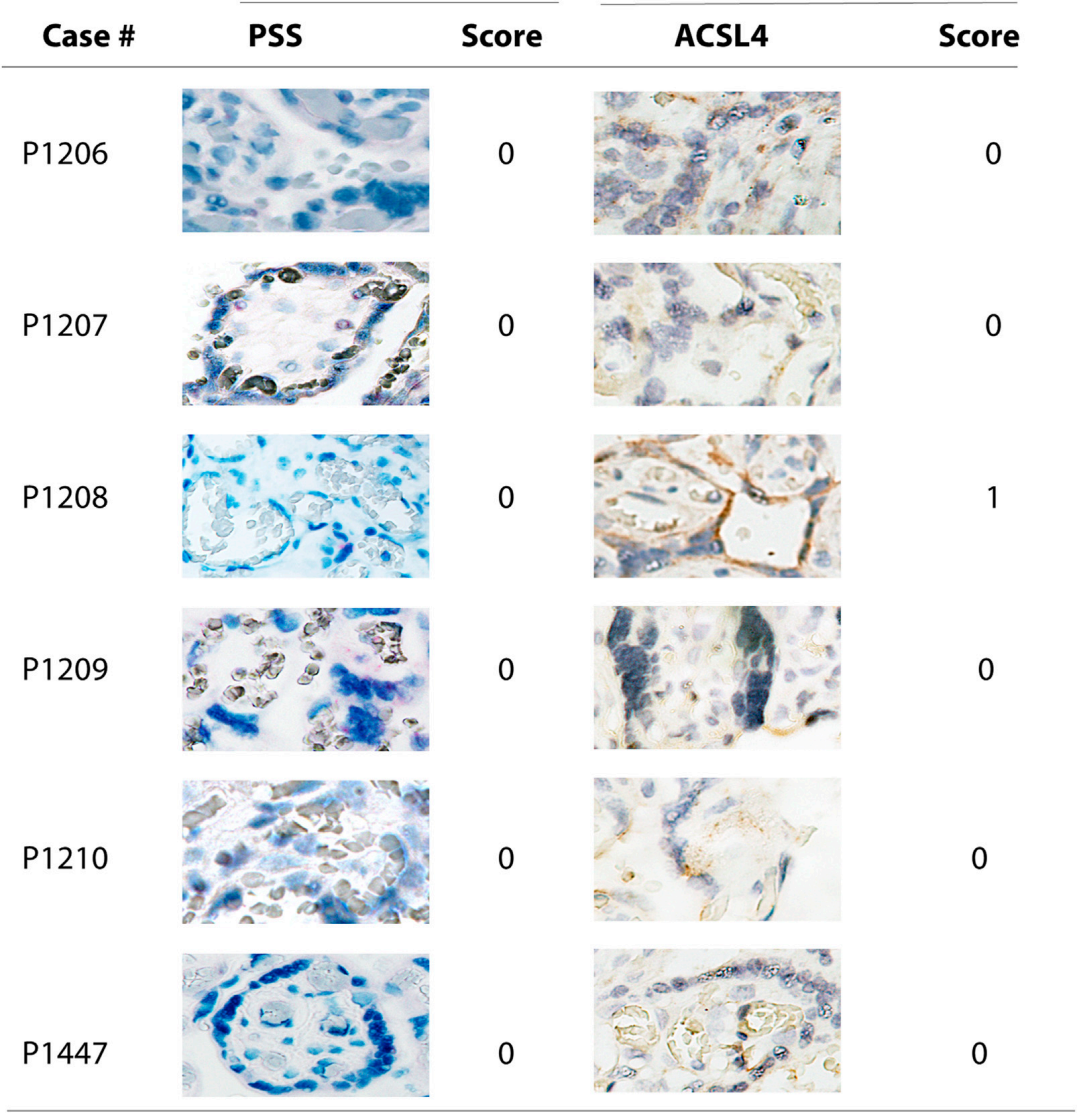
Microscope images (20X) of viral RNA and ACSL4 protein staining in placental villous tissues among specimens with negative PSS staining. RNA-ISH with use of RNAScope HD-RED kit and immunohistochemical staining (IHC) were performed to determine the abundance of the viral RNA genome and ACSL4 protein, respectively.

Combined with the data showing that no significantly positive staining of either PSS or ACSL4 was observed in all six negative cases (Figure 4), these results further support that SARS-CoV-2 is a potent inducer for activation of ferroptosis in the human placenta.

### A positive trend between SARS-CoV-2 infection degree and ACSL4 expression levels

**FIGURE 5:**
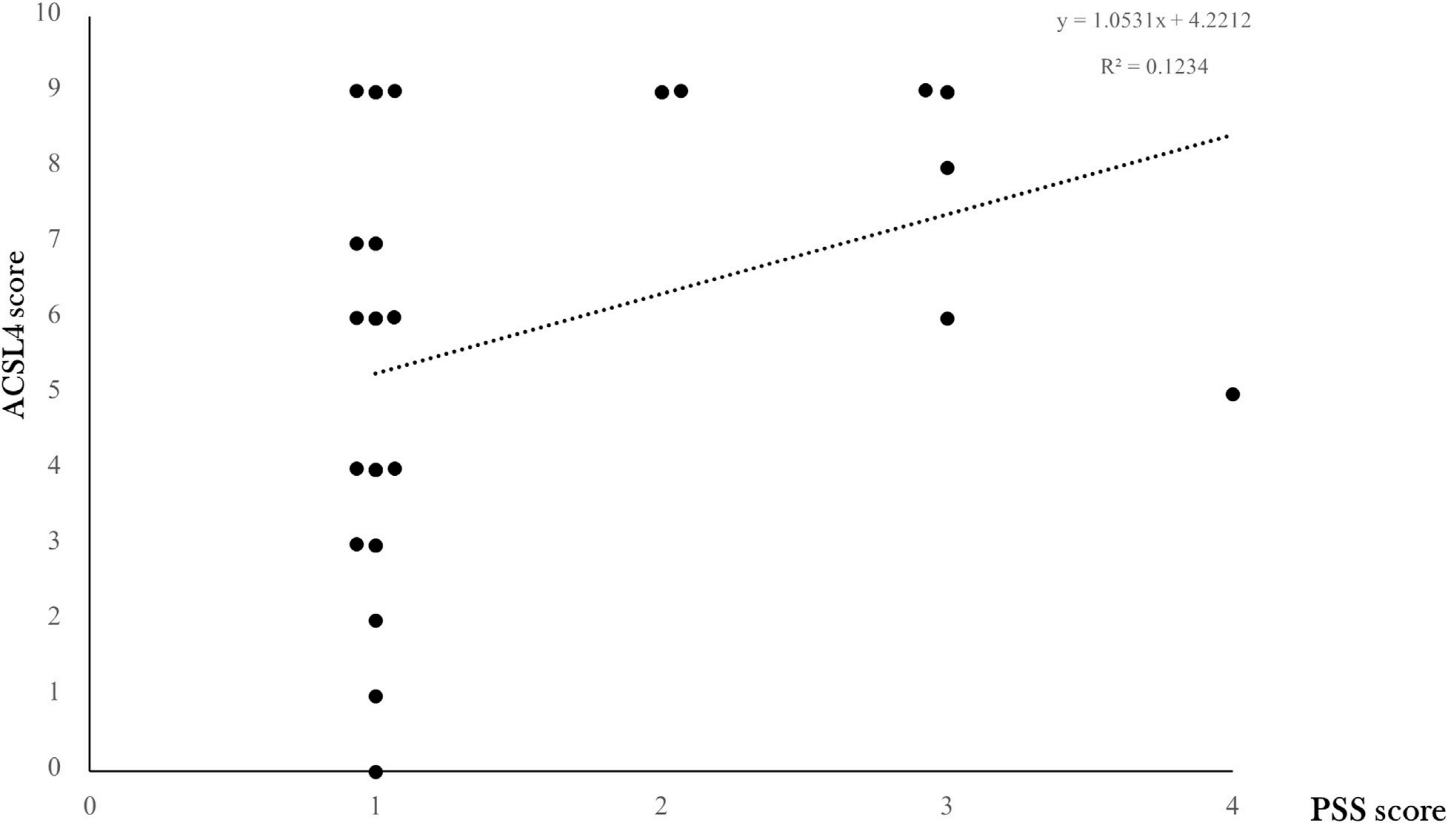
ACSL4 expression levels according to placental SARS-CoV-2 infection degree. The correlation between ACSL4 expression levels and overall viral PSS staining score was modeled by linear regression with a positive trend (y = 1.0531x +4.2212, *R* ^2^ = 0.1234) shown in a dotted line.

Despite no significance observed, a linear regression analysis showed a positive trend (y = 1.0531x +4.2212, R2 = 0.1234) of increased scoring of PSS staining along with increased ACSL4 expression levels (Figure 5).

## Discussion

The findings of this study are twofold. On the one hand, we used RNAScope-based RNA-ISH assay, a newly developed ISH platform, which has been extensively used for the detection of viral infection in vertebrate tissues (van der Eijk et al., 2016; Smith et al., 2019; Liu et al., 2020), to examine the PSS abundance in placentas collected from pregnant females tested positive for SARS-CoV-2 infection. The strengths of the RNAScope ISH platform primarily result from its unique double-Z probe design, which permits a superior background control with much less nonspecific hybridization (Wang et al., 2012). We found that 23 specimens demonstrated a convincingly positive staining of PSS in placental chorionic villi tissues including syncytitrophoblast, cytotrophoblast, and fetal blood vessels to varying extents, in contrast to all six placentas collected from normal pregnant females tested negative for SARS-CoV-2, which showed no signs or signals for infection whatsoever. On the other hand, in these 23 specimens we further found that ACSL4 expression levels were significantly increased compared with those of six negative counterparts, and correlation analysis revealed a positive trend existing between staining degree of PSS and ACSL4. As a result, our study not only provides robust evidence for capacity possessed by SARS-CoV-2 virions to infect human placenta, but also supports that ferroptosis is activated in the response to this viral infection.

Our study has shown that SARS-CoV-2 can function as a potent trigger for activation of ferroptosis in human placenta. A variety of infectious agents including viruses can influence production of reactive oxygen species, iron metabolism and transport, and antioxidative defenses to a substantially great scope (Stockwell, 2022). Patients with COVID-19 demonstrated higher serum ferritin levels, consistent with an elevated iron exposure in tissues (Kaushal et al., 2022). In addition, lipids alteration and induction of transferrin receptor, another well investigated and specific biomarker for ferroptosis (Feng et al., 2020), have been reported in Syrian golden hamsters following a challenge by of SARS-CoV-2 virions (Bednash et al., 2022). ACSL4, typical marker of ferroptosis owing to its capability to preferentially activate long-chain polyunsaturated fatty acids (PUFAs) for phospholipid biosynthesis and fueling ferroptotic process, has been considered as the major determinant for dictating ferroptosis sensitivity and reshaping cellular lipid composition (Doll et al., 2017), which are highly susceptible to peroxidation because of the presence of extraordinarily weak C-H bonds existing between adjacent C=C double bonds (Stockwell, 2022). Mechanistically, by virtue of its role in facilitating incorporation of PUFAs into membrane lipids, ACSL4 is also capable of amplifying the ferroptosis process in a feed-forward mechanism characterized with the loop of protein kinase C beta 2-phosphorylated ACSL4 (Zhang et al., 2022). To this end, ACSL4 is unanimously deemed as an essential executioner of ferroptosis, comparable to the role of caspase-3 as in apoptosis.

Placental susceptibility to ferroptosis highlights a key role of a major cascade of molecular events linking initiation of a pathophysiological condition resulting from SARS-CoV-2 crossing of the placental barrier to ultimate manifestations of pregnancy-related sequalae. Indeed, human placenta can produce a wide array of hormones and cytokines, such as human chorionic gonadotropin and progesterone, to sustain pregnancy, and therefore, placental dysfunction largely accounts for pathogenesis of most adverse pregnancy outcomes. A recent systematic review and meta-analysis on the impact of COVID-19 on pregnancy outcomes involving 42 studies and 438,538 patients concluded that SARS-CoV-2 infection is closely associated with risks of hypertension disorders of pregnancy, intrauterine fetal demise, and preterm birth (Wei et al., 2021). In addition, SARS-CoV-2 infection has been shown to be associated with spontaneous abortion in a cohort study of 3,041 pregnancies conducted in the United Kingdom (Balachandren et al., 2022). Nonetheless, elevated levels of oxidative stress and subsequent accumulation of reactive oxygen species and lipid peroxidation have been frequently implicated in placental insufficiency and related diseases (Schoots et al., 2018; Zhang et al., 2020). An excessive accumulation of hydroperoxy-arachidonoyl (C20:4)- or adrenoyl (C22:4)- phosphatidylethanolamine (Hp-PE) characterizes ferroptotic cell death, and ferroptosis proceeds when Hp-PE levels exceed cellular defense (Kagan et al., 2017; Anthonymuthu et al., 2018). By using mass spectrometry-based phospholipidomics analysis, a recent study by Beharier and others has found that preterm birth-associated injured placentas exhibited augmented levels of Hp-PE (Beharier et al., 2020). Combined with these published studies, our study further supports that the role of ferroptosis in molecularly connecting SARS-CoV-2 infection and adverse obstetrical outcomes deserves further investigations.

Some limitations are associated with this study. Because of limited sample size, we were not able to reach a conclusion with respect to a significant trend positively correlating the degree of placental SARS-CoV-2 infection and scopes of ferroptosis activation. Furthermore, the lack of *in vitro* including use of primary cytotrophoblast as the model as well as *in vivo* animal studies makes it difficult to establish a definitive causal relationship. However, these experiments will be included in our future directions.

In summary, this study has uncovered that SARS-CoV-2 infection may activate ferroptosis in the human placenta. Thus, pregnant females diagnosed with COVID-19 need to be considered at high risks for adverse clinical outcomes and should be included in all preventive strategies. Moreover, our study provides opportunities to ultimately control and manage adverse pregnancy-related outcomes, which can be facilitated by development of potent and selective chemical compounds and biologics based on specifically targeting markers characteristics of ferroptosis in the placenta. As such, ferroptosis inhibition could constitute a viable strategy to prevent placental injury and insufficiency due to viral infection in the clinical care setting.

## Data Availability

The original contributions presented in the study are included in the article/supplementary material further inquiries can be directed to the corresponding authors.
